# Sustained benefits of cognitive training in children with inattention, three-year follow-up

**DOI:** 10.1371/journal.pone.0246449

**Published:** 2021-02-04

**Authors:** Barbora G. Jurigova, Molly R. Gerdes, Joaquin A. Anguera, Elysa J. Marco

**Affiliations:** 1 Department of Neurology, University of California San Francisco, San Francisco, California, United States of America; 2 Helen Wills Neuroscience Institute, University of California Berkeley, Berkeley, California, United States of America; 3 Research Division, Cortica Healthcare, San Rafael, California, United States of America; 4 Department of Psychiatry, University of California San Francisco, San Francisco, California, United States of America; Universtiyt of Oviedo (Spain), SPAIN

## Abstract

The goal of this study was to test for long-term benefits three years after the completion of a cognitive training intervention (Project: EVO^™^) in a subset of children with Sensory Processing Dysfunction (SPD). Our initial findings revealed that children with SPD who also met research criteria for Attention Deficit Hyperactivity Disorder (SPD_+IA_) showed a significant decrease in parent-observed inattentive behaviors, which remained stable in a nine-month follow-up assessment. Forty nine caregivers of participants who completed the Project: EVO^™^ training were contacted to be included in this follow up study. Each was emailed an invitation to complete the Vanderbilt ADHD Diagnostic Parent Rating Scale, which yielded a completion rate of 39/49 (80%). A Generalized Estimating Equations analysis was used to assess changes in symptoms over time, specifically to determine whether the initial improvements were retained. The SPD_+IA_ cohort continued to show sustained benefits on their parent-reported scores of inattention, with 54% of SPD_+IA_ individuals no longer meeting criteria for ADHD three years following intervention. These findings provide initial insights into the potential long-term benefits of a digital health intervention for children with attention-based issues.

## Introduction

Sensory Processing Dysfunction (SPD), expressed as exaggerated aversive, withdrawal, or seeking behaviors associated with sensory inputs [1], affects almost five percent of all children [2]. Even though SPD is categorized as atypical responses to sensory input, many of those affected also exhibit attentional challenges [1, 3]. These findings suggested that a robust assessment and subsequent intervention of these deficient cognitive abilities may be warranted in this particular population. Research along these lines from our group has indeed demonstrated that compared to their typically developing peers, approximately 40% children with SPD demonstrate diminished cognitive control (selective attention in particular) as well as visuomotor control—abilities crucial to academic achievement and social development [4, 5]. In addition, these individuals also show decreased white matter microstructural integrity that correlated with their issues of inattention [6], providing a structural suggestion as to why such inattention effects may be present.

The use of digital technologies, especially those with adaptive mechanics, to assessing cognitive control have been especially powerful to help contend with inherently elevated testing variability in different clinical populations [5, 7]. Such approaches also underlie the basis for many digital therapeutic interventions: our work with the NeuroRacer intervention demonstrated that a ‘video game’ with adaptive mechanics can lead to improved attention abilities both behaviorally and neurally in older adults, with such effects persisting 6 months beyond the training period [8]. Out of the NeuroRacer platform came Project: EVO^™^, a digital intervention designed to enhance cognitive control abilities, specifically attention and goal management [5, 9]. Project: EVO was modified into an iOS compatible platform that deploys modern videogame interface with engaging visual and auditory feedback and adaptive algorithms designed to constantly challenge each player’s abilities. The effectiveness of Project: EVO was supported by improvements in cognitive control in different populations, including children with Autism Spectrum Disorder (ASD) and Attention Deficit Hyperactivity Disorder (ADHD) as well as older adults with geriatric depression [7, 9–11]. Most recently, a randomized, controlled trial found Project:EVO, currently known as EndeavorRx, to significantly improve attention in a large sample of children with ADHD [12]. Based on those results, The U.S Food and Drug Administration granted EndeavorRx^™^ (AKL-T01) clearance to be prescribed as a treatment for children with ADHD.

Previous study data published in 2017 provided promising evidence for cognitive benefits of Project: EVO in a subset of children with SPD who experience greater cognitive control deficiencies (SPD+IA) compared to typically developing children (TDC) evidenced by poorer neural and behavioral performance on measures of attention [5]. These SPD_+IA_ individuals showed improvements in parent-reported attention following EVO training, with the improvements persisting at a 9-month follow-up. In contrast, SPD children without attention difficulties and TDC did not demonstrate training related benefits. In addition to the decrease in ADHD symptoms, participants in the SPD_+IA_ group showed increased midline frontal theta power, a neural marker of attention [13–16]. These improvements correlated with the parental reports of inattention improvement. The 2017 study demonstrated the potential of Project: EVO to significantly improve attention after 4 weeks of training in children with SPD who experience attentional challenges. While findings from the original study are promising, it was not clear whether these benefits would persist over a longer time period than the 9-month follow-up period. Indeed, a number of longitudinal studies have shown persistence of benefits for several years [17, 18], but such effects and their duration following a behavioral intervention in the SPD population are unknown. The purpose of the current follow-up study is to assess whether the parent-reported benefits observed after EVO training in the SPD_+IA_ group were sustained at a 3-year follow-up.

## Materials and methods

### Participants

Participant recruitment for the pilot study took place between February 2014 and January 2015 from the Sensory Neurodevelopment and Autism Program at University of California, San Francisco (UCSF). This follow-up study was conducted between March and May of 2018 involving 49 participants from the original cohort who successfully completed EVO training. Out of those, parents of 39 participants (80%) responded to this three-year follow-up, which involved the Vanderbilt ADHD Diagnostic Parent Rating Scale. Participants’ caregivers were contacted via email and completed the study through REDCap, a secure online platform for consenting and data collection. UCSF’s IRB committee approved this study through IRB #10–01940. An electronic written consent was obtained from all participants.

### Measures

#### Vanderbilt ADHD Diagnostic Parent Rating Scale (Vanderbilt)

Participants’ caregivers were administered the Vanderbilt scale [19, 20] prior to the intervention as well as at each follow-up session to assess observed changes in participants’ attention and behavior. The Vanderbilt scale was shown to have an excellent internal consistency reliability (0.90 ≤ α ≤ 0.95) as well as high concurrent validity (r = 0.79; [21]) when related to the Computerized Diagnostic Interview Schedule for Children (C-DISC-IV; National Institute of Mental Health, 1997) [22]. The Vanderbilt scale assesses the 18 symptoms of ADHD as outlined in the DMS-IV [23]. The Vanderbilt scale was also utilized to distinguish two subgroups within the SPD cohort: those that reached the standardized threshold for inattention or hyperactivity (SPD+IA) and those that did not (SPD). The first follow-up assessment took place shortly after a participant completed 20 days of training with a second follow-up 9 months later [5]. The present study assesses the retainment of improvements three years post intervention. Parents were administered the first 18 questions of the Vanderbilt scale, where the first 9 questions assess symptoms of Inattention and the other 9 questions assess Hyperactivity/Impulsivity on a scale from 0 (“Never”) to 3 (“Very often”). To meet the criteria for either Inattention or Hyperactivity/Impulsivity, scores of 2 or 3 must be selected for at least 6 out of the 9 items. For quantitative analyses, sum from both scales were calculated as well as a total score combining both scales.

### Project: EVO training

The Project: EVO^™^ (EVO) is proprietary software developed by Akili Interactive Labs, specifically designed as an investigational medical device to assess and adaptively train cognitive control for populations with cognitive disorders or decline and executive function deficits. EVO is a self-guided treatment designed for at-home use that involves a combination of visuomotor and perceptual discrimination tasks played on an iPad. Each session of the EVO training lasts approximately 4 minutes and consists of a multitasking condition, during which participants perform the visuomotor and perceptual discrimination tasks simultaneously [5]. The EVO intervention program involved participants playing 7 sessions (~30 minutes), 5 times a week for the duration of 1 month (20 days total). Research assistants remotely monitored EVO play and provided support and feedback to the parents and children during training. If a research assistant noticed a participant had more than two incomplete days of training, a reminder phone call would be made to the parents.

### Statistical analyses

Age and gender comparability of the current groups (SPD, SPD_+IA_ and TDC) were assessed with one-way ANOVA and chi-square test, respectively. Considering that the sample of individuals participating in this follow-up was 20% smaller than the original sample, we also checked for significant differences between our subsample and those who did not fill out the follow-up questionnaire. Specifically, age and baseline inattention contrasts were assessed using independent samples t-tests and gender representation with a chi-square test.

Improvements in Inattention were analyzed using Generalized Estimating Equations (GEE) with robust standard error estimators. GEE is a regression-based method used for analyzing longitudinal data where observations across time are not independent [24]. Since Inattention is a continuous variable, we selected Gaussian distribution with identity link function. The main goal of this study was to determine whether children in the SPD_+IA_ group sustained the improvements observed after EVO. Thus, the first GEE model assessed changes over time within the SPD_+IA_ group with baseline as the reference. Additionally, the interaction of Group and Time was assessed in a separate model with SPD_+IA_ group as the reference for the Group variable and baseline scores as the reference of time.

All statistical analyses were performed using the SPSS 20.0 (IBM SPSS Statistics).

## Results

Out of the 49 contacted participants, 39 parents (80%) completed the Vanderbilt 3-year follow-up assessment. The SPD, SPD_+IA_ and TDC groups did not differ significantly in terms of age (*p* = .326) or gender (*p* = .283). Additionally, the subsample involved in the current follow-up was comparable to the original sample analyzed in the pilot study in terms of age, gender and baseline Vanderbilt Inattention scores with one exception in the TDC group—those who participated in this follow-up (M = 6.37, SD = 3.06) had a significantly higher or more impaired (*p* = .042) baseline inattention scores compared to those who did not (M = 2.33, SD = 2.31; Table 1).

**Table 1:** Sample characteristics.

	TDC		SPD		SPD_+IA_		Total	
	*Pilot*	*FU*	*Pilot*	*FU*	*Pilot*	*FU*	*Pilot*	*FU*
N	22	19	10	7	17	13	49	39
Males	12 (55%)	10 (53%)	8 (80%)	6 (86%)	10 (59%)	7 (54%)	30 (61%)	23 (59%)
Mean Age (SD)	14.32 (1.1)	14.47 (1.1)	14.8 (1.6)	14.57 (1.5)	13.65 (1.5)	13.77 (1.7)	14.18 (1.4)	14.26 (1.4)
Baseline Inattention (SD)	5.82 (3.25)	6.37 (3.06)*	13.5 (2.07)	14.14 (2.12)	20.94 (3.11)	20.69 (3.15)	12.63 (7.38)	12.54 (7.11)

Sample characteristics of the original cohort (Pilot) and the subsample involved in the current follow-up study (FU).

*statistically significant at *p*≤0.05

To assess the long-term effects of the Project: EVO training on parental observations of inattention, we compared the Vanderbilt scores at 4 points in time (pre-assessment, post-assessment, 9-month and 3-year follow-up) for each of the 3 groups (SPD, SPD_+IA_, TDC). Mean total scores as well as mean scores from each subscale (Inattention, Hyperactivity) at all points in time are presented in Table 2. When setting up the GEE model, several choices need to be made. First, a working correlation matrix that best represents the relationships between measurements at different time points needs to be identified. Initially, autoregressive correlation matrix was considered based on a trend toward diminishing correlations over time. However, this type of correlation matrix assumes equal time intervals between any two observations [25], therefore exchangeable correlation matrix was chosen in the end. One of the advantages of GEE is that it is considered robust against misspecified correlation matrices [26]. The results of the GEE analysis (Table 3) revealed that the Inattention scores of the SPD_+IA_ group at each follow-up assessment (post intervention, 9 months, and 3 years later) were significantly lower relative to baseline. This supports our hypothesis that the improvements of Inattention observed in children with SPD_+IA_ immediately after intervention were sustained 3 years later. Notably, 7 (54%) out of the 13 SPD_+IA_ individuals no longer met clinical criteria (scores 2 or 3 on at least 6 out of 9 items) for Inattention at 3-year follow-up.

**Table 2:** Changes in Vanderbilt mean scores.

	**Vanderbilt TOTAL Mean (SD)**			
	**PRE**	**POST**	**9M**	**3Y**
TDC	9.82 (5.16)	9.00 (3.99)	7.62 (5.31)	6.16 (7.18)
SPD	23.80 (5.20)	23.20 (8.89)	19.00 (6.02)	19.00 (12.32)
SPD_+IA_	37.65 (5.31)	31.06 (6.77)	29.50 (9.11)	26.23 (10.88)
	**Vanderbilt INATTENTION Mean (SD)**			
	**PRE**	**POST**	**9M**	**3Y**
TDC	5.82 (3.25)	5.75 (2.69)	4.76 (3.60)	4.16 (4.40)
SPD	13.50 (2.07)	12.70 (4.27)	10.13 (2.85)	12.86 (8.51)
SPD_+IA_	20.94 (3.11)	16.63 (3.50)	16.00 (4.50)	14.38 (5.64)
	**Vanderbilt HYPERACTIVITY/IMPULSIVITY Mean (SD)**			
	**PRE**	**POST**	**9M**	**3Y**
TDC	4.00 (2.80)	3.25 (2.88)	2.86 (2.61)	2.00 (3.22)
SPD	10.30 (3.97)	10.50 (5.19)	8.88 (3.72)	6.14 (4.78)
SPD_+IA_	17.71 (5.17)	14.44 (4.59)	13.50 (6.74)	11.85 (6.43)
N	49	46	45	39

Vanderbilt mean scores at 4 time points: pre-assessment (PRE), post-assessment (POST), 9-month follow-up (9M), and 3-year follow-up (3Y).

**Table 3:** GEE analysis of Inattention scores.

	**Estimated change**	**SE**	**95% CI**	
SPD_+IA_ change from baseline				
POST	-4.44***	1.12	-6.64	-2.25
9M	-5.16***	1.1	-7.3	-2.98
3Y	-6.59***	1.57	-9.66	-3.52
	**Group comparisons**			
	**Estimated difference**	**SE**	**95% CI**	
SPD vs. SPD_+IA_				
POST	3.64*	1.5	0.7	6.59
9M	1.64	1.41	-1.12	4.39
3Y	5.38	2.94	-0.37	11.13
TDC vs. SPD_+IA_				
POST	4.29**	1.22	1.9	6.69
9M	3.94**	1.44	1.12	6.77
3Y	4.84***	1.81	1.29	8.38

Results of the GEE analysis performed on the Vanderbilt Inattention scores collected at 4 time points: baseline, post intervention (POST), 9-month follow-up (9M), and 3-year follow-up (3Y). First third of the table shows changes in scores within the group of children with Sensory Processing Dysfunction and Inattention (SPD+IA) relative to baseline. The rest of the table shows estimated differences between those changes and changes observed in the other two groups: typically developing children (TDC) and children with Sensory Processing Dysfunction only (SPD).

SE, Standard error; CI, Confidence interval.

*p<0.05,

**p<0.01,

***p<0.001

**Fig 1:**
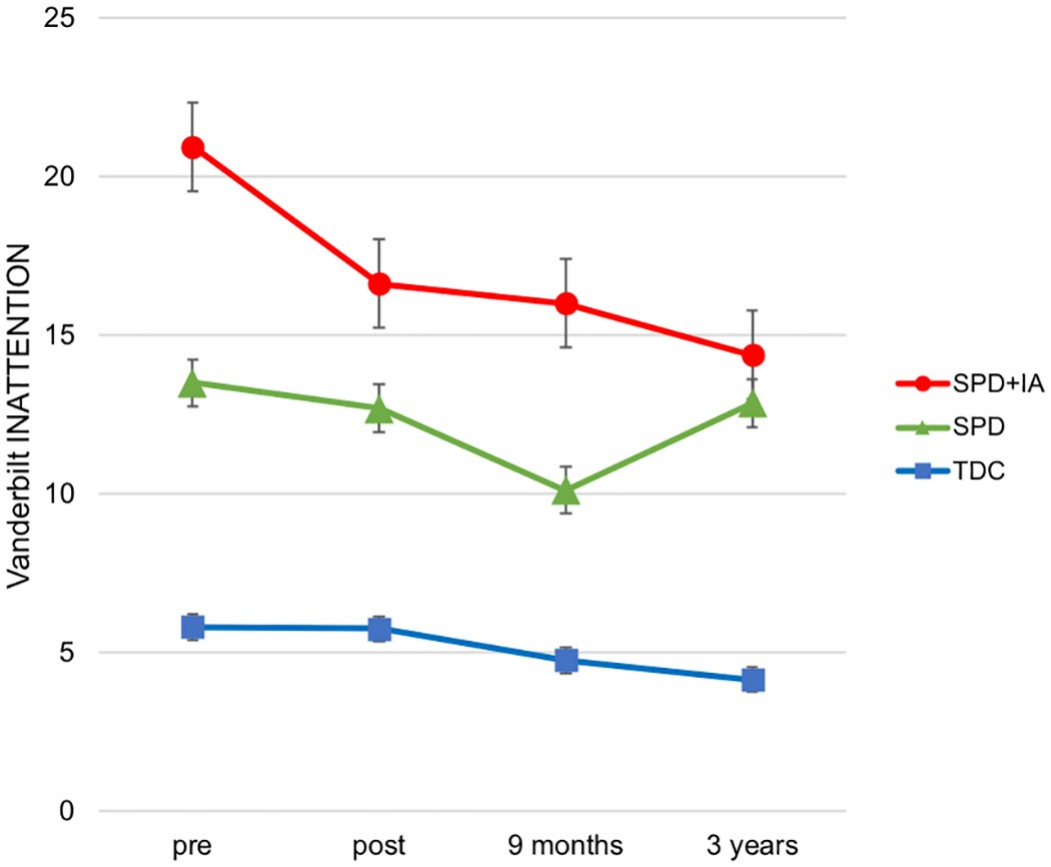
Changes in Vanderbilt Inattention scores. Vanderbilt Inattention mean scores displayed separately for typically developing children (TDC), children with Sensory Processing Dysfunction only (SPD), and children with SPD combined with inattention (SPD_+IA_) groups at 4 time points: pre-assessment [N = 49, N(TDC) = 22, N(SPD) = 10, N(SPD_+IA_) = 17], post-assessment [N = 46, N(TDC) = 20, N(SPD) = 10, N(SPD_+IA_) = 16], 9-month follow-up [N = 45, N(TDC) = 21, N(SPD) = 8, N(SPD_+IA_) = 16], and 3-year follow-up [N = 39, N(TDC) = 19, N(SPD) = 7, N(SPD_+IA_) = 13]. Error bars indicate standard error of the mean.

As can be seen in Fig 1, the scores of all three groups decreased over time to some degree. Thus, we also examined the interaction of Group and Time, in order to determine whether the changes in symptoms in SPD_+IA_ were different from the changes observed in typically developing children and SPD children without attentional challenges. The interaction of Group and Time was statistically significant (*p* = .017). Inattention scores of the TDC group at the 3-year follow-up changed from baseline by approximately -6.59 + 4.84 = -1.75 points, which was a significantly smaller decrease in symptoms compared to the one seen in children in the SPD_+IA_ group (*p* = .000445). The scores of the SPD-only group changed from baseline by approximately -6.59 + 5.38 = -1.21 over the 3-year period. While the difference in changes between the SPD and SPD_+IA_ group was not statistically significant (*p* = .067), possibly because of a smaller sample size of the SPD-only group at the 3-year follow-up (N = 7), the actual difference in changes was greater than the difference between the TDC and SPD_+IA_ group (4.84<5.38).

In addition to the Inattention scale, we conducted GEE analyses on the Hyperactivity/Impulsivity and Total scores. The interaction of Group and Time was not statistically significant in the analysis of Hyperactivity/Impulsivity scores (*p* = .114) and any changes within the SPD_+IA_ group did not differ significantly from those observed in the other two groups. Outcomes of the Vanderbilt Total scale, which is a combination of the two subscales, were comparable to those of Inattention. These results are presented in S1 Table.

## Discussion

The purpose of this study was to assess the long-term retention of parent reported inattention-based improvements following digital intervention with Project: EVO in children with Sensory Processing Dysfunction (SPD). Using a parent report, we found that children with SPD who also experience attentional challenges (SPD+IA) sustained their improvements three years post intervention and the majority of these children no longer meet clinical criteria for inattention. Below we discuss the implications of these findings with respect to targeted cognitive remediation in this cohort, as well as limitations that need to be considered.

Numerous studies have found beneficial effects of cognitive training in substitution or in addition to pharmacological treatments in children with attentional challenges [27–42] as well as in healthy children [43]. A handful of those studies followed-up with their participants to see whether any observed changes persisted over time, ranging from 6 weeks to 1 year after training [34–37, 39, 41, 43]. To our best knowledge, this is the longest follow-up study assessing long-term effects of cognitive training in children as well as the first study of this kind in children with SPD.

Our findings of long-lasting improvements in attention among SPD children with ADHD symptoms are consistent with previous reports of children diagnosed with ADHD showing sustained cognitive and behavioral benefits months after interventions [34–37, 39]. For example, Rabiner et al. [41] found that only children with six or more symptoms of inattention based on DSM-IV criteria demonstrated persistent benefits of computer-based attention training a year later. Taken together, these findings provide support for potential long-term benefits of cognitive training in children with attentional difficulties.

While it is difficult to determine the primary reason(s) for the sustained benefits in our SPD_+IA_ cohort, we can draw from previous work to propose plausible explanation. The premise of cognitive training programs is that controlled engagement in cognitive tasks with adaptive algorithms provides constant challenge thus leading to strengthening of brain networks and enhancing the corresponding cognitive functions [44]. This hypothesis was recently corroborated by an fMRI study of children with ADHD [42] that demonstrated that cognitive training led to increased task-related activation in right dorsolateral prefrontal cortex (DLPFC) as well as inferior and superior parietal regions. The DLPFC along with parieto-temporal regions, cerebellum, thalamus and basal ganglia, are all brain areas considered to play an important role in mediating sustained attention, and that have been previously found to be underactivated in children with ADHD [45].

Taking into account the considerable symptom overlap and possible common neuroanatomical factors in ADHD and SPD [46, 47], the hypothesis of mechanism of change induced by cognitive training in ADHD may also be relevant in our SPD_+IA_ cohort. Electroencephalography (EEG) data from our pilot study [5] showed significantly lower midline frontal theta (MFT) power, a known neuromarker of attention [8, 13, 14], in the SPD_+IA_ group when compared to typically developing children (TDC). Following EVO training, MFT increased significantly in SPD_+IA_ and reached levels comparable to those observed in the TDC group at baseline. Moreover, the MFT power gain correlated positively with improvements on the Vanderbilt Inattention scale. This regional theta activity is thought to reflect the communication between basal ganglia and frontal regions [48], brain areas involved in the sustained attention network.

These findings suggest that cognitive training might stimulate neural circuitry under-activated in children with attentional challenges, thus helping them “get on track” and resulting in sustained improvements years after intervention. Normalization effects have previously been observed for other modes of intervention, specifically pharmacological treatment. Structural and functional neuroimaging studies found that brain regions involved in modulating attention, including right DLPFC, basal ganglia, and cerebellar vermis, reached normal levels in long-term stimulant medicated ADHD patients [49–52]. This points to the possibility that cognitive training acts on these neural regions in a similar way as psychostimulant medication. To our best knowledge, neuroimaging data supporting long-term effects of cognitive training are not yet available, which stresses the importance of including neural assessment in future studies.

The clearest limitation of the present study is its sole reliance on parent report questionnaires. In the future, we hope to assess each participant not just on a parent report basis, but obtain neural data for the same assessments that were completed in the initial study. These data would aid in establishing whether the changes in neural activity observed in SPD_+IA_ at post-assessment were also preserved three years later and whether they correlated with behavioral data reported by parents.

The absence of a control group is another notable limitation of this study. Looking at data from all 4 time points, we observed a pattern of symptom reduction, to varying degrees, among all 3 groups, which could be accounted for by the process of maturation. Even though the changes observed within the SPD_+IA_ group were significantly greater than changes measured within the other 2 groups, having a sex- and age-matched control group of SPD_+IA_ children without exposure to Project EVO, would shed more light into the specific contribution of this cognitive intervention program on the reported behavioral changes.

## Conclusions

Despite the limitations, this follow-up study provides promising evidence for long-term benefits of Project: EVO in a subset of children with SPD. These children experience attention deficits that often impact their academic and social development. Our results demonstrate that just four weeks of videogame-like cognitive training have the potential to improve symptoms of inattention in this group of SPD children and that these improvements are sustained years after intervention with the majority of these children no longer meeting clinical criteria for Inattentive subtype of ADHD. Further studies are needed, however, to verify these results with the inclusion of control groups as well as multifaceted assessments.

## Supporting information

S1 Table GEE analysis of Vanderbilt hyperactivity/impulsivity and total scores. (DOCX)

S1 File Dataset. Individual Vanderbilt scores at all measurement times.
(XLSX)
