# Supplementary material for: Sustained benefits of cognitive training in children with inattention, three-year follow-up
Source: PLoS One. 2021 Feb 4;16(2):e0246449. doi: 10.1371/journal.pone.0246449 (PMC7861383; doi:10.1371/journal.pone.0246449)
Supplement: S1 Table — (DOCX) [file pone.0246449.s001.docx]

**S1 Table. GEE analysis of Vanderbilt Hyperactivity/Impulsivity and Total scores**

| HYPERACTIVITY / IMPULSIVITY | | | | | TOTAL | | | | |
| --- | --- | --- | --- | --- | --- | --- | --- | --- | --- |
|  | **Estimated change** | **SE** | **95% CI** | |  | **Estimated change** | **SE** | **95% CI** | |
| SPD_+IA_ change from baseline | | | | | SPD_+IA_ change from baseline | | | | |
| POST | -2.37** | 0.79 | -3.91 | -0.82 | POST | -6.82*** | 1.2 | -9.17 | -4.47 |
| 9M | -3.15** | 1.21 | -5.52 | -0.78 | 9M | -8.31*** | 1.98 | -12.19 | -4.43 |
| 3Y | -5.18*** | 1.49 | -8.11 | -2.25 | 3Y | -11.75*** | 2.52 | -16.69 | -6.82 |
|  | **Group comparisons** | | | |  | **Group comparisons** | | | |
|  | **Estimated difference** | **SE** | **95% CI** | |  | **Estimated difference** | **SE** | **95% CI** | |
| SPD vs. SPD_+IA_ | | | | | SPD vs. SPD_+IA_ | | | | |
| POST | 2.57 | 1.67 | -0.7 | 5.83 | POST | 6.22* | 2.55 | 1.22 | 11.22 |
| 9M | 1.6 | 1.77 | -1.86 | 5.06 | 9M | 3.26 | 2.46 | -1.57 | 8.08 |
| 3Y | 0.05 | 2.17 | -4.19 | 4.3 | 3Y | 5.4 | 4.57 | -3.55 | 14.34 |
|  |  |  |  |  |  |  |  |  |  |
| TDC vs. SPD_+IA_ | | | | | TDC vs. SPD_+IA_ | | | | |
| POST | 1.53 | 0.93 | -0.29 | 3.35 | POST | 5.82*** | 1.48 | 2.93 | 8.72 |
| 9M | 1.99 | 1.36 | -0.66 | 4.65 | 9M | 5.95* | 2.43 | 1.19 | 10.71 |
| 3Y | 2.96 | 1.71 | -0.39 | 6.31 | 3Y | 7.78** | 2.99 | 1.91 | 13.65 |
| Results of the GEE analysis performed on the Vanderbilt Hyperactivity/Impulsivity (left) and Vanderbilt Total (right) scores collected at 4 time points: baseline, post intervention (POST), 9-month follow-up (9M), and 3-year follow-up (3Y). First third of the table shows changes in scores within the group of children with Sensory Processing Dysfunction and Inattention (SPD_+IA_) relative to baseline. The rest of the table shows estimated differences between those changes and changes observed in the other two groups: typically developing children (TDC) and children with Sensory Processing Dysfunction only (SPD). | | | | | | | | | |
| SE, Standard error; CI, Confidence interval. | | | | |  | | | | |
| **p*<0.05, **p<0.01, ****p*<0.001 | |  |  |  |  |  |  |  |  |
